# Investigating Bacteriophages Targeting the Opportunistic Pathogen *Acinetobacter baumannii*

**DOI:** 10.3390/antibiotics9040200

**Published:** 2020-04-22

**Authors:** Kathryn M. Styles, Rapee Thummeepak, Udomluk Leungtongkam, Sophie E. Smith, Gabrielle S. Christie, Andrew Millard, John Moat, Christopher G. Dowson, Elizabeth M. H. Wellington, Sutthirat Sitthisak, Antonia P. Sagona

**Affiliations:** 1 School of Life Sciences, University of Warwick, Coventry CV4 7AL, UK; Kathryn.Styles@warwick.ac.uk (K.M.S.); sophieelizabethsmith94@gmail.com (S.E.S.); j.moat@warwick.ac.uk (J.M.); C.G.Dowson@warwick.ac.uk (C.G.D.); E.M.H.Wellington@warwick.ac.uk (E.M.H.W.); 2 Department of Microbiology and Parasitology, Faculty of Medical Science, Naresuan University, Muang, Phitsanulok 65000, Thailand; rapee_worm32@hotmail.com (R.T.); undelete_nahm@hotmail.com (U.L.); Sutthirats@nu.ac.th (S.S.); 3 School of Biosciences, University of Birmingham, Birmingham B15 2TT, UK; GXC460@student.bham.ac.uk; 4 Department of Genetics and Genome Biology, Leicester University, Leicester LE1 7RH, UK; adm39@leicester.ac.uk; 5 Warwick Antimicrobial Screening Facility, University of Warwick, Coventry CV4 7AL, UK

**Keywords:** *Acinetobacter baumannii*, antibiotic resistance, Thailand, opportunistic, nosocomial, bacteriophage, antibiotic alternative, phage therapy

## Abstract

The multi-drug resistance of the opportunistic pathogen *Acinetobacter baumannii* is of growing concern, with many clinical isolates proving to be resistant to last resort as well as front line antibiotic treatments. The use of bacteriophages is an attractive alternative to controlling and treating this emerging nosocomial pathogen. In this study, we have investigated bacteriophages collected from hospital wastewater in Thailand and we have explored their activity against clinical isolates of *A. baumannii*. Bacteriophage vB_AbaM_PhT2 showed 28% host range against 150 multidrug resistant (MDR) isolates and whole genome sequencing did not detect any known virulence factors or antibiotic resistance genes. Purified vB_AbaM_PhT2 samples had endotoxin levels below those recommended for preclinical trials and were not shown to be directly cytotoxic to human cell lines in vitro. The treatment of human brain and bladder cell lines grown in the presence of *A. baumannii* with this bacteriophage released significantly less lactate dehydrogenase compared to samples with no bacteriophage treatment, indicating that vB_AbaM_PhT2 can protect from *A. baumannii* induced cellular damage. Our results have also indicated that there is synergy between this bacteriophage and the end line antibiotic colistin. We therefore propose bacteriophage vB_AbaM_PhT2 as a good candidate for future research and for its potential development into a surface antimicrobial for use in hospitals.

## 1. Introduction

*Acinetobacter baumannii* infection has become a research priority due to its exceptional ability to acquire antimicrobial resistance (AMR) [1,2]. Strains of this Gram negative bacterium have been shown to have high genetic diversity and large genetic ‘resistance islands’ [3], and there have been cases of pan-drug resistant isolates that are resistant to all known clinical antibiotics [4]. This has made *A. baumannii* a formidable pathogen in the nosocomial environment, particularly against immunocompromised and intensive care unit (ICU) patients. Often targeting moist tissues such as mucous membranes [5], *A. baumannii* infection will commonly result in urinary tract infections, meningitis, pneumonia, and wound site infections. Forming strong biofilms, *A. baumannii* is also commonly connected with the use of catheters and ventilators [6], the latter of which is associated with particularly high mortality rates [7]. 

Current therapies against *A. baumannii* infections include the beta-lactamase inhibitor sulbactam, carbapenems, aminoglycosides, and tetracyclines [8]. However, there is no longer an empirical antimicrobial agent treatment [8] and the extensive use of the broad spectrum carbapenems [9] has led the World Health Organization to highlight carbapenem resistant *A. baumannii* as a Class 1: Priority Critical pathogen for the focus of research and development of novel antibiotics in 2017 [1]. End line treatment options include tigecycline and colistin [10], both of which have been associated with severe side effects, including hepatotoxicity, nephrotoxicity, and neurotoxicity [6].

*A. baumannii* infections are of particular concern in Thailand [11] where this pathogen is the most common cause of death by AMR associated nosocomial infection [7]. According to NARST (National Antimicrobial Resistance Surveillance Centre, Thailand), 98% of *A. baumannii* isolates were susceptible to the carbapenem imipenem in 1998 [1,12], but by 2002, this had dropped to 79%, and by 2008 only 43% of isolates screened were susceptible. Data collected for 2018 indicated that just over 32% of *A. baumannii* isolates in Thailand were susceptible to imipenem and 31.8% to meropenem. In the same year, only 30.7% of 42,212 isolates were susceptible to sulbactam, 39.1% to the aminoglycoside gentamycin, and 18.5% to tetracycline [12]. Currently, 97.1% of isolates are recorded to be susceptible to the last resort treatment, colistin. However, resistance genes to this antibiotic were discovered in 2011 [13] and if the trends follow those seen for the carbapenems, the number of colistin susceptible *A. baumannii* isolates could be reduced by two thirds within the next 20 years.

Bacteriophages are an attractive alternative to treating this drug resistant pathogen in the upcoming ‘post antibiotic era’ [14]. The application of lytic bacteriophages could be prepared as a cocktail or as a combinatorial therapy, thereby reducing the chances of novel resistances being developed [15]. Phage therapy [16] has been shown to be successful in treating mouse models for *A. baumannii* pneumonia [17] and wound infections [18], with the first human trial of bacteriophages being used against *A. baumannii* published in 2017 [19]. Gaining regulatory approval for phage therapy is complex and slow however, and as yet, there are no approved mainstream bacteriophage medicines in the EU or USA. In 2007 though, the Food and Drug Agency (FDA) approved the use of bacteriophages as a food additive to protect consumers against *Listeria monocytogenes* in meat [20], and over time, the regulation of phage therapy may become more straight forward. Until this time, bacteriophages still have the potential to be utilised for the benefit of human health in hand sanitizers, surface disinfectants, or antimicrobials [21], and should not be overlooked as a powerful disease control tool. We are only just beginning to touch on the scope of how bacteriophages could be used in the pursuit of substantial human health benefits.

This study initially investigated five bacteriophages, isolated from hospital wastewater treatment plants in the lower northern Thai province of Phitsanulok, due to their good host range against 150 multidrug resistant (MDR) *A. baumannii* isolates [11,22]. A preliminary investigation using 24-h growth curves indicated that bacteriophage vB_AbaM_PhT2 not only had the highest relative host range of those screened, but also maintained low *A. baumannii* turbidity for the longest period of the five isolates during growth curves. This bacteriophage was therefore carried forward for a more detailed investigation, with the aim of potentially developing a surface disinfectant. Upon sequencing of the vB_AbaM_PhT2 genome, there were no known virulence factors or antibiotic resistance genes found. The treatment of human brain and bladder cell lines with this bacteriophage produced a significant reduction in relative cytotoxicity caused to the human cells by being grown in the presence of *A. baumannii*. There was also an indication of vB_AbaM_PhT2 synergy with colistin, potentially allowing the clinical dosage of this nephrotoxic drug to be reduced. We therefore propose vB_AbaM_PhT2 as a candidate for future research and development into a tailored surface antimicrobial for use in hospitals.

## 2. Results

### 2.1. Acinetobacter baumannii Bacteriophages Present Broad Host Range

**Figure 1:**
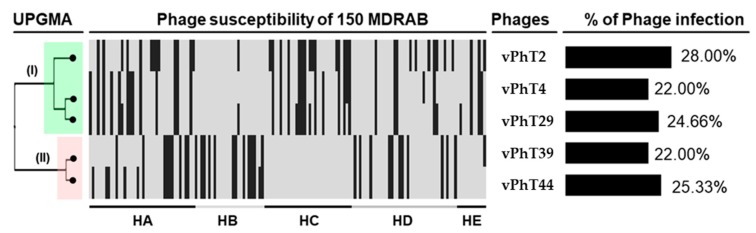
Information on host ranges of Thai bacteriophages against 150 multidrug resistant *Acinetobacter baumannii* (MDR-AB) strains from five hospitals. From left to right are presented: a UPGMA (Unweighted Pair Group Method with Arithmetic mean) dendrogram, host susceptibility to isolates from different hospitals (black = *A. baumannii* isolates susceptible to bacteriophages (clear plaque), grey = *A. baumannii* isolates resistant to the bacteriophages (no plaque), HA–HE = hospitals A–E) and bacteriophage names and percentage host ranges.

Bacteriophages, previously isolated from wastewater at Buddhachinaraj and Bang Rakam hospitals, were screened against multidrug resistant (MDR) *A. baumannii* strains [22] from five tertiary hospitals (HA-HE, see methods) (where MDR-AB is defined as strains resistant to at least three classes from penicillins/cephalosporins, fluroquinolones, and aminoglycosides [23,24]). The five of these bacteriophages with the highest host range were carried forward for further investigation in this study (Table 1 and Figure 1). All five bacteriophages had been previously partially characterised and were all confirmed to belong to the lytic non-enveloped dsDNA *Caudovirales* order of viruses via electron microscopy [25,26,27,28,29] (Supplementary Figure S2). Bacteriophages vPhT2, 4, and 44 were all *Myoviridaes*, whereas vPhT29 and 39 were the smaller *Podoviridaes*. Formerly, these bacteriophages were published under a different, less standardised nomenclature, but have now been renamed to reflect the Kropinski system [30] (see methods) for clarity and consistency with other bacteriophage research (Table 1). These bacteriophages are now called vB_AbaM_PhT2, vB_AbaM_PhT4, vB_AbaP_PhT29, vB_AbaP_PhT39, and vB_AbaM_PhT44 (previously ØAB02, ØAB04, ØAB29, ØAB39 and ØAB44), but for the purposes of the flow of the text in this paper, these names have also been abbreviated to vPhT2, vPhT4, vPhT29, vPhT39, and vPhT44 respectively [30]. 

This study showed host ranges of 28.00%, 22.00%, 22.00%, 24.66%, and 25.33% when screened against 150 MDR *A. baumannii* for vPhT2, vPhT4, vPhT29, vPhT39, and vPhT44, respectively. Ninety (60%) of the 150 isolates were killed by at least one of the five bacteriophages. An unweighted pair group method with arithmetic mean (UPGMA) dendrogram (Figure 1), which was created to represent the binary clustering of susceptibility of the 150 MDR-AB isolates to the bacteriophages, revealed that the five bacteriophages could be grouped into two distinct clusters (clusters I and II), based on similarities in host range. Cluster I consisted of three bacteriophages (vPhT2, vPhT4, and vPhT39), whereas vPhT29 and vPhT44 belonged to cluster II.

### 2.2. Bacteriophage Activity against Their Bacterial Host

#### 2.2.1. Bacteriophages Reduce *A. baumannii* Turbidity over 24 Hours

**Figure 2:**
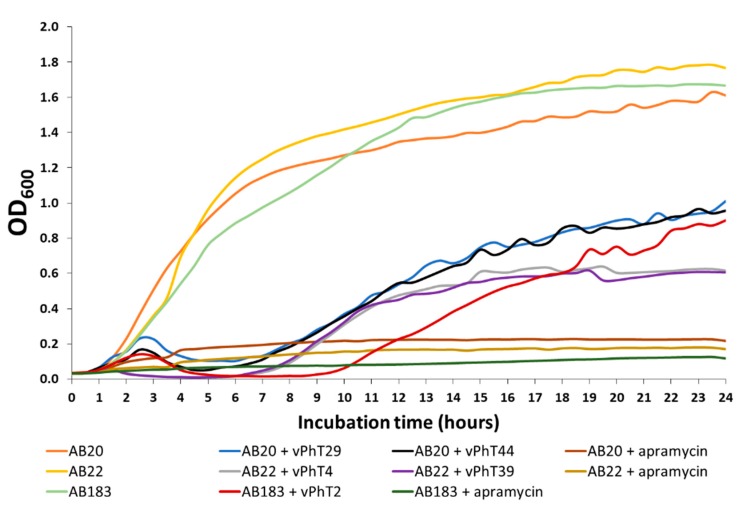
Twenty-four-hour growth curve for *Acinetobacter baumannii* strains grown with and without bacteriophages or apramycin (500 µg/mL). Data displayed as OD_600_ (N = 3).

In a 24-h growth curve of bacterial samples treated with each bacteriophage at an MOI of 0.01, there was a brief rise in OD_600_ (up to ~1.5 to 2.0 h incubation) before a drop in turbidity compared to the untreated controls (Figure 2). For bacteriophage vPhT44, the OD_600_ remained low until around 5.5 h, when it gradually increased again for the rest of the time course. This gradual increase was also seen for vPhT4, vPhT29, and vPhT39 from around 6.5 h, but for vPhT2, the increase was not until around nine hours. The OD_600_ of all samples being treated with bacteriophages remained well below that of the untreated controls throughout the 24-h incubation period (Table 2). The lowest OD_600_ readings over the 24-h period (not including lag phase readings during the first hour) after treatment with vPhT2, vPhT4, vPhT29, vPhT39, and vPhT44 were observed after 7.0, 4.5, 6.0, 4.5, and 4.5 h respectively (Figure 2).

Samples of *A. baumannii* were also treated with apramycin to produce a comparison of changes in cell turbidity when treated with known antibiotics versus bacteriophages. The turbidity of the samples treated with apramycin remained low throughout the time course with the lowest OD_600_ being produced much earlier than the bacteriophage samples, at around 1.5 h, after which there was a slow but gradual increase.

#### 2.2.2. Bacteriophages Reduce Relative *A. baumannii* Metabolic Activity over 24 Hours

As a further assessment of relative bacteriophage activity against their host, an endpoint triphenyl tetrazolium chloride (TTC) assay was performed on growth curve cultures, where the colorimetric TTC product was a measure of dehydrogenase activity and cell respiration, and was proportional to bacterial metabolic activity (measured at OD_540_) (Table 2). A significant reduction in metabolic activity (as well as turbidity) was observed for all bacteriophages at 24 h compared to the untreated controls (where significance was calculated using a two-tailed, paired *t*-test with a *p*-value of less than 0.05 being defined as significant). The percentage reduction in relative *A. baumannii* metabolic activity (OD_540_) ranged between 26.27% and 41.04% and turbidity (OD_600_) was reduced between 39.16% and 67.04%. Bacteriophage vPhT4 produced the greatest reductions in both metabolic activity and turbidity by 24 h. There was a positive correlation coefficient between turbidity and metabolic activity for all samples at 24 h (*r* = 0.95, *p* < 0.00001).

#### 2.2.3. Pairs of Bacteriophages Do Not Act Synergistically

Growth curves were also produced using mixes of pairs of bacteriophages that had the same host (vPhT4 and vPhT39 with AB22 and vPhT29 and vPhT44 with AB20). As before, the relative endpoint metabolic activity (TTC at OD_540_) and turbidity (OD_600_) of the host *A. baumannii* were analysed (Table 2). No synergy was detected and so the use of these combinations was not replicated in future testing.

#### 2.2.4. Bacteriophage vPhT2 Presents the Best Performance in all the Parameters Tested

No single bacteriophage performed best in every single assay performed. The relative performance of bacteriophages across all assays was compared in order to select one for further characterisation (Table 3). Bacteriophage vPhT2 performed consistently well across all experiments: it maintained a low *A. baumannii* OD_600_ for the longest period in the 24-h growth curves (Figure 2) and had the widest host range (Figure 1). Bacteriophage vPhT2 was therefore selected for an investigation of its suitability as a surface antimicrobial or as a potential candidate to be used for phage therapy.

### 2.3. Genomic Analysis of vPhT2

#### 2.3.1. Sequencing of the Genome of vPhT2 Phage

**Figure 3:**
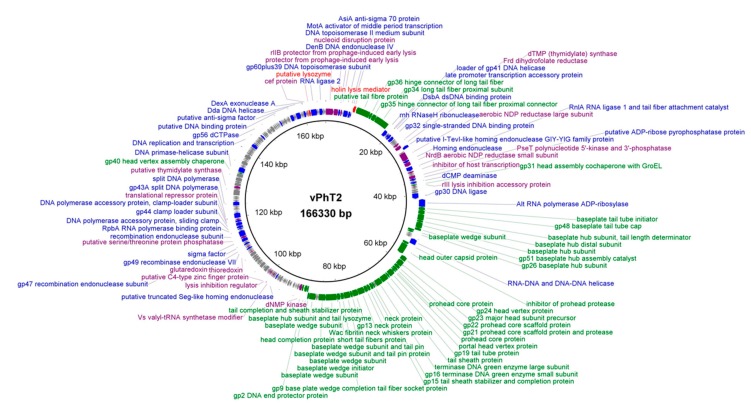
Graphic circular map of vPhT2 genome and its predicted open reading frames (ORFs) (NCBI accession number: MN864865). From inside to outside: the scale units in kilo base pairs (kbp), predicted genes on forward strand and predicted genes on reverse strand. Predicted genes are coloured according to their function (green = structural and morphogenesis proteins, blue = DNA replication and transcription, red = host lysis, purple = other functions and grey = hypothetical proteins with unknown function). The genome map was drawn using the BLAST Ring Image Generator (BRIG) software, version 0.95, The University of Queensland, Brisbane, Australia.

Sequencing of the vPhT2 genome revealed a genome size of 166,330 bp, with 246 predicted protein coding sequences and 9 tRNAs in a single chromosome with an average coverage of 596x (Figure 3, Table 4) (NCBI accession number: MN864865). The genome was determined to be double-stranded DNA with a G + C content of 36.4%. Of the 246 predicted open reading frames (ORFs), the majority genes encoded for proteins with no known function (144 genes) and 102 putative gene products (41%) were assigned to known functions. Putative proteins encoded by vPhT2 can be categorised into four groups: structural and morphogenesis (43 proteins), DNA replication/transcription (37 proteins), host lysis (2 proteins), and ‘other’ (20 proteins) (Figure 3). The major capsid proteins, major and minor tail proteins, baseplate hub subunit protein, tail fiber proteins and prohead core proteins involved in structure and morphogenesis were identified, as were the genes involved in DNA replication and transcription, such as endodeoxyribonucleases, DNA helicases, DNA polymerases, transcriptional regulators, and RNA ligases. With regards to host lysis by vPhT2, an endolysin and holin lysis mediator were found. Moreover, a gene coding for a tail-associated lysozyme was found (locus tag: vB_AbaM_PhT2_173). Additional genes identified coded for a predicted thymidylase synthase, DNA adenine methylase, acetyltransferase, thymidylate synthase and dihydrofolate reductase (Figure 3). No known genes involved in antibiotic-resistance and virulence factors were detected in the vPhT2 genome when searched in the virulence factor database (VFDB) [31] and in the antibiotic resistance genes database (ARDB) [32].

#### 2.3.2. Comparison of vPhT2 Genome against the NCBI Database

A BLASTn [33] search against the NCBI database using vPhT2 genome as a query resulted in the identification of the five most closely related genomes, AbTZA1, AM101, KARL-1 [34], vB_ApiM_fHyAci03 [35] and ZZ1 [36] (Table 4), all of which were *Acinetobacter* infecting bacteriophages. At the nucleotide level, the vPhT2 genome was only genetically *very* similar to Israeli isolate AbTZA1, where an average nucleotide identity (ANI) of 95.34% was calculated (with a cut-off of >95% to represent being of the same species). A low ANI (~71–78%) was seen with the next four most similar bacteriophages, namely AM101, KARL-1, vB_ApiM_fHyAci03, and ZZ1.

### 2.4. Preparations of Bacteriophage vPhT2 Shows Good Stability in Long Term Studies in Lysogeny Broth, but Not in SM Buffer II

**Figure 4:**
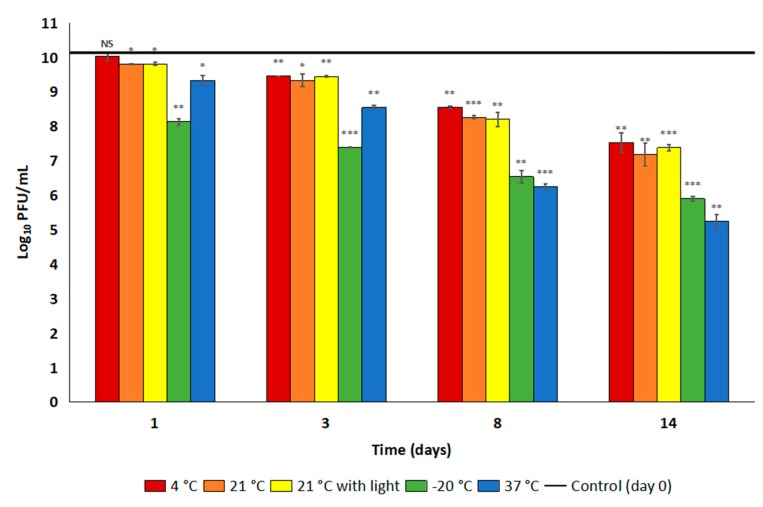
Logarithmic titre of vPhT2 after storage in SM buffer II or LB for 14 days in different temperature and light conditions compared to day zero. (NS = no significance, * *p* < 0.05, ** *p* < 0.01, *** *p* < 0.001).

Aliquots of bacteriophage vPhT2 stored in lysogeny broth (LB) or SM buffer II (see methods) at a number of temperatures and in the presence and absence of light were titred at multiple recovery points in order to establish the laboratory stability of the suspensions. Bacteriophage suspensions stored in LB in the fridge (4 °C) and at room temperature (21 ± 2 °C), both protected from and exposed to light, did not show a significant reduction in titre during the first 14 days of storage (Table 5 and Supplementary Table S2). When the samples were stored in SM buffer II however, a reduction in titre was seen almost immediately with reductions of 1.57 ± 0.03, 1.87 ± 0.04 and 1.92 ± 0.21 log_10_PFU/mL after only eight days at 4 °C, 21 °C without light, and 21 °C with light respectively (Figure 4). This degradation continued over the rest of the time course, with a greater than 2.5 log reduction being seen after 14 days storage for all three samples. When stored in LB in the fridge during an extended study over 10 months, only a 1.30 ± 0.08 log_10_PFU/mL reduction in bacteriophage titre was seen in this time (data not shown here). When comparing the room temperature samples (21 °C) kept in the dark with those exposed to light, there was no significant difference in titre of samples stored in SM buffer II seen at any time point for 14 days (*p* > 0.05).

For samples stored in SM buffer II in the freezer (−20 °C), there was already a 1.99 ± 0.09 log_10_PFU/mL reduction in titre after only 24 h, compared to time zero (*p* < 0.05) (Table 5). This then followed with log reductions of 2.74 ± 0.01, 3.59 ± 0.18 and 4.23 ± 0.06 log_10_PFU/mL by three (3), eight (8) and fourteen (14) days storage compared to time zero. Storage at 37 °C in SM buffer II resulted in the greatest degradation of vPhT2, with a significant reduction in titre of 0.79 ± 0.14 log_10_PFU/mL seen after 24 h (*p* < 0.05). By three days this was a 1.58 ± 0.06 log_10_PFU/mL reduction and by eight and 14 days this was 3.88 ± 0.07 and 4.88 ± 0.21 log_10_PFU/mL, respectively.

### 2.5. vPhT2 Activity in the Human Cell Environment

#### 2.5.1. CsCl Purified vPhT2 Preparations Had Low Endotoxin Levels

Bacteriophage vPhT2 was purified via a caesium chloride gradient prior to testing in the human cell environment (and stability testing trials) in order to minimise the number of endotoxins in the preparation. A 1 × 10^8^ PFU/mL sample of CsCl purified vPhT2 in SM buffer II was found to have 10.92 endotoxin units per millilitre (EU/mL) (Table 6). Crude lysates containing vPhT2 in LB had endotoxin levels above the threshold of the assay.

#### 2.5.2. vPhT2 Presents No Cytotoxicity to Human Cell Lines

Lactate dehydrogenase is an enzyme found in nearly all living cells and catalyses the production of pyruvate from lactate and back again, producing NAD^+^ and NADH [37]. As an intracellular enzyme, the extracellular presence of LDH in a supernatant is an indicator of cytotoxicity, human cell line death and damage. Treatment of T24 and hCMEC/D3 cell lines with bacteriophage vPhT2 (in the absence of *A. baumannii*) produced no significant difference (*p* > 0.05) in LDH release compared to cell lines that had not been treated, indicating that this bacteriophage was not cytotoxic (Table 7). All human cell lines had a significant increase (*p* < 0.05) in levels of LDH released after treatment with *A. baumannii* (in the absence of bacteriophages) compared to no bacterial infection (Table 7), indicating that the presence of *A. baumannii* was cytotoxic.

#### 2.5.3. vPhT2 Reduces LDH Production by Human Cell Lines Grown in the Presence of *A. baumannii*

**Figure 5:**
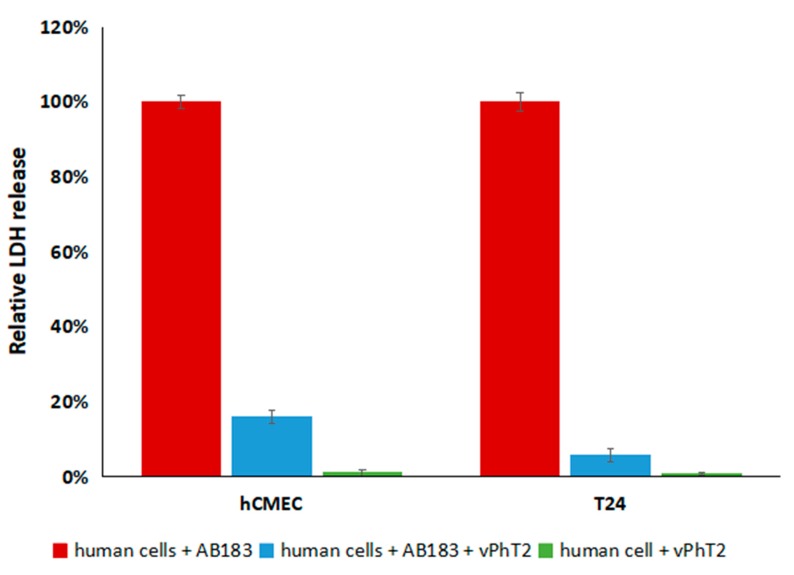
Normalised lactate dehydrogenase levels (LDH) (OD_490_–OD_680_) released by T24 and hCMEC/D3 human cell lines treated with *Acinetobacter baumannii* AB183 and or vPhT2 over 24 h (N = 3). Treatment of human cells with only AB183 represent 100% relative cytotoxicity. LDH levels produced by human cell negative controls with no treatment are representative of 0% cytotoxicity.

When vPhT2 was applied to human cell lines that had been exposed to *A. baumannii* for an hour, there was a 90.98 ± 0.49% and 84.01 ± 1.74% reduction in LDH production over the next 23 h by T24 and hCMEC/D3 cells, respectively (*p* < 0.001) (Figure 5). 

#### 2.5.4. vPhT2 Reduces *A. baumannii* Activity in the Presence of Human Cell Lines

As a marker of bacterial inhibition, the endpoint turbidity (OD_600_) and titres of *A baumannii* from samples of the LDH assay were measured. The reduction in OD_600_ for the supernatant of the human cell lines exposed to *A. baumannii* and vPhT2 was 85.19% ± 4.85% and 89.35% ± 1.72% for T24 and hCMEC/D3 cells respectively, compared to samples only treated with *A. baumannii* (Table 8) (*p* < 0.001). There were also measured to be log reductions in *A. baumannii* titre of 3.34 ± 0.11 and 3.08 ± 0.13 log_10_CFU/mL when treated with vPhT2 in the presence of T24 and hCMEC/D3 cells respectively (Table 8). Unsurprisingly, there was a positive correlation between relative LDH levels produced by the human cell lines and the reduction in *A. baumannii* OD_600_, (*r* = 0.52, *p* < 0.00001) or endpoint titre (*r* = 0.67, *p* < 0.001). As *A. baumannii* growth was reduced, so were the levels of LDH released by human cell lines.

### 2.6. vPhT2 Works Synergistically with Colistin

Host strain *A. baumannii* AB183 was shown to be susceptible to colistin (EUCAST clinical breakpoint >2 µg/mL [38]) (Supplementary Table S3 and Figure S4) with a measured MIC of 1 µg/mL. Previous studies have indicated that endolysins from *A. baumannii* bacteriophages can work synergistically with colistin [27]. When colistin and bacteriophage vPhT2 were tested in combination against AB183, a fractional inhibitory concentration index (FICI) value of 0.35 was calculated (Table 9), indicating synergy between this bacteriophage and colistin (where the FICI was interpreted as follows: FICI ≤ 0.5, synergy; <2 and >0.5, indifference; ≥2, antagonism [39]).

## 3. Discussion

The five bacteriophages in this study had a host range of between 22% and 28% of 150 MDR *A. baumannii* isolates. When analysing host range data and discussing the ‘broadness’ of the range, the diversity of location sources of the hosts should be taken into consideration [40]. Bacteriophages isolated from hospital waste-water are more likely to be efficacious against clinical isolates from the same hospital or region than independent sources, due to coevolution [28,29]. The *A. baumannii* isolates screened in this study were from five hospitals spread across Thailand, a country of over 500,000 km^2^ with a population of around 70 million inhabitants [41]. Hospitals were chosen to be representative of a range of different environments including industrial and rural areas, international borders, the presence of tourists and foreign workers, as well as geographic locations and regional climates. All five bacteriophages were from two hospitals in lower northern Thailand. Thus, the bacteriophages appear to have the potential to be used in a range of Thai hospital locations and possibly the rest of the world. In fact, a screen of the NCBI database [33] showed vPht2 to be of the same species (% ANI > 95) as a bacteriophage isolated from Israel, AbTZA1 (accession number: MK278860). Groupings produced by the UPGMA dendogram, also revealed that there are two groups of bacteriophage host ranges within the five bacteriophages screened. If applied as a cocktail of cluster I (vPhT2, vPhT4, vPhT39) and II (vPhT29 and vPhT44), the overall host ranges could be expected to be higher [29]. In fact, 60% (90 out of 150) of the clinical isolates were killed by at least one of the five bacteriophages. 

Over the course of a 24-h growth curve, the OD_600_ of samples treated with the bacteriophages did not increase past 60.84% of the negative controls. Although there was a significant reduction in host turbidity by all bacteriophages at all-time points analysed, bacteriophage vPhT4 produced the greatest relative reductions in *A. baumannii* metabolic activity (TTC assay) and turbidity (OD_600_), whereas bacteriophage vPhT2 maintained the low OD_600_ for the longest period (nine hours compared to 5.5–6.5 h for all others). Bacteriophage vPhT2 also had the highest host range and for this reason was selected for further investigation and characterisation.

The production of sequencing data on vPhT2 means there is now the option for the genetic engineering of this bacteriophage, which could potentially lead to the optimisation of its use for multiple commercial applications. An analysis of the vPhT2 genome showed that it contained the gene to carry a lysozyme domain fused to its tail, suggesting that this bacteriophage encodes enzymes capable of degrading host’s cell wall at the phage’s first contact with the cell surface and the last step of the phage lytic cycle [42]. As the genome did not harbour any known virulence and antibiotic resistance genes that could potentially confer to a host, it shows promise as a suitable candidate for further applications in phage therapy. 

Stability and a sensible shelf-life are important for the accessibility and usefulness of an antibacterial product in the clinical environment. Any specific storage conditions could complicate existing hospital procedures and possibly lead to incorrect usage by untrained individuals. It is promising therefore that the vPhT2 titre was stable at room temperature, the most likely routine storage temperature for a disinfectant, when stored in LB (both in the presence and absence of light) for 14 days. After 10 months in the fridge in this same diluent, there was just over a one log reduction in vPhT2 titre, indicating that this is a possible long-term storage option. However, lysogeny broth is not a common commercial storage medium for bacteriophages and it is unclear why the bacteriophage was less stable in SM buffer II. Further investigation is therefore required to find a more suitable, commercially relevant diluent for vPhT2. We also suggest the use of an opaque storage vessel in order to protect the long-term efficacy of bacteriophage suspensions.

Endotoxins are part of the outer membrane of the cell wall of Gram negative bacteria and are a known pyrogenic contaminant of injectable drugs [42]. Endotoxin levels for purified vPhT2 (1 × 10^8^ PFU/mL) were shown to be below the threshold of 200 EU/mL as proposed by Brito et al. for endotoxin levels in live attenuated vaccines going through preclinical trials, based on United States Pharmacopoeia (USP) advice [42,43]. Should a higher dose or volume be required, endotoxin levels for dosages up to almost 20 times higher would still be below these guideline amounts and well within the USP recommendation of 5 EU/kg/hr [44].

The treatment of human T24 bladder and hCMEC/D3 brain cell lines with *A. baumannii* and vPhT2 resulted in a significant reduction in cytotoxicity (as measured by relative LDH levels) compared to when no bacteriophage was present. In addition, neither human cell line grown in the presence of vPhT2 (with no *A. baumannii*) released significantly more LDH than controls grown without exposure to bacteriophages indicating that vPhT2 is not cytotoxic.

Previous trials with purified *A. baumannii* bacteriophage endolysins showed broad range host activity and synergy with colistin [27]. The polymyxin colistin, with all its associated side effects, is one of the end line treatments for *A. baumannii* infection. With an FICI of 0.35, the indication that colistin can work synergistically with bacteriophage vPhT2 is very promising. Developing vPhT2 for use in the clinical environment, for example through developing an encapsulated delivery system [45] or further stability trials are therefore to be considered as the next steps for bacteriophage vPhT2.

## 4. Materials and Methods 

### 4.1. Collection of Samples and Host Range Analysis

Seventeen bacteriophages were isolated from wastewater at Buddhachinaraj and Bang Rakam hospitals in Phitsanulok province, Thailand in 2010 and were screened against 150 MDR *A. baumannii* strains collected from five hospitals [11,22,27,29] using a spot test method as described previously [27]. For additional information on the chosen bacteriophages and MDR-AB, please refer to the supplement file: ‘Selected-MDRAB-150-isolates’. The first hospital (HA) was located in central Thailand, another one was in the lower northern region (HB), another one was on the northern tourist border with Myanmar (HC), one was located in an eastern industrial area with a high population of foreign workers (HD) and the final hospital was in northern Thailand (HE). Each hospital had between 405 and 1000 beds at the time of the study and isolates were collected from a range of different departments and specimen types.

Colonies of *A. baumannii* were suspended in 0.85% NaCl to an equivalent of a 0.5 McFarland standard (1 × 10^8^ CFU/mL). The suspension was then swabbed on to Trypticase Soy Agar (TSA) to create a bacterial lawn. Phage suspensions (2 µL at 1 × 10^8^ PFU/mL) were dropped into the lawn before plates were incubated at 37 °C for eight (8) hours to allow cell lysis. Bacterial clearance at the site of bacteriophage inoculation implied that the host was sensitive to that particular bacteriophage. All experiments were performed in duplicate.

Of the five bacteriophages with the highest host range, bacteriophages vPhT4 and vPhT39 were tested against the chosen *A. baumannii* host strain AB22, vPhT29 and vPhT44 against the host strain AB20 and vPhT2 against the host strain AB183 (Table 1, Supplementary Figure S1 and Table S1) due to the good titres seen for these bacteriophage-host pairs in initial screening studies. Bacteriophage vPhT2 also showed some specificity against host strain AB22. Bacteriophages vPhT2, 4, 39 and 44 were all isolated from Buddhachinaraj hospital whereas vPhT29 was from Bang Rakam hospital. Hosts for propagating these bacteriophages were isolated from two different hospitals, one in northern Thailand and another in central Thailand (Supplementary Table S1) and cultivated in LB and LBA (lysogeny broth agar) (1.5% agar) as described previously [29].

#### 4.1.1. Analysis of Host Range Data

A UPGMA dendrogram was created using DendroUPGMA: a dendrogram construction utility website using the default settings [46]. The input query was for a binary outcome (yes or no). If a particular bacteriophage could infect an *A. baumannii* host, then it was given a rating equal to one (yes), and if it could not infect, it was given a rating equal to zero (no).

#### 4.1.2. Naming of the Bacteriophages

The five bacteriophages were renamed (Table 1) for this study according to Kropinski system [30] to make their referencing and entry into open access databases more consistent with contemporary and progressive research. The prefix of vB_AbaM_PhT or vB_AbaP_PhT was used to indicate that these were bacterial viruses (vB), infecting *A. baumannii* (Aba), were either *Myoviridaes* or *Podoviridaes* (M or P) and were isolated from Phitsanulok province in Thailand (PhT). This nomenclature system was not used in previous publications with these bacteriophages as they were first isolated only a year after the Kropinski system was first suggested in 2009 and knowledge of this nomenclature was not widespread or commonly practiced.

### 4.2. Bacteriophage Characterisation

#### 4.2.1. Viral Enrichment—Propagation of Bacteriophages of Interest

To propagate the bacteriophage isolates, the *A. baumannii* hosts were grown overnight in LB (Sigma-Aldrich: Lennox—10 g/L tryptone, 5 g/L yeast extract, 5 g/L NaCl) at 37 °C and 130 rpm. In the morning, 1 mL of the overnight liquid culture was used to inoculate 50 mL fresh LB. This was then incubated at 37 °C and 130 rpm until an OD_600_ of 0.3 was reached. At this point, 100 μL of bacteriophage stock was added to each flask and samples were incubated for a further four hours. Bacterial debris was pelleted by centrifugation at 3220 g for 10 min before the supernatant was passed through a 0.2 μm pore size membrane filter. The prepared phage stocks in LB were stored at 4 °C. Bacteriophage samples stored in LB were used for initial 24-h growth curve assays, stability trials, and other bacterial studies.

#### 4.2.2. Caesium Chloride Purification of Bacteriophages

Bacteriophages were propagated as described above, with the assay being scaled up to a 250 mL sample. Sodium chloride was added to samples to a final concentration of 1 M. After incubation on ice for one hour, samples were centrifuged at 3220× *g* and the supernatant passed through a 0.2 μm pore size membrane filter before PEG8000 was added to a final concentration of 10% *w*/*v*. This was then left overnight at 4 °C, before the samples were centrifuged at 25,000× *g* for 60 min. The phage pellet was then resuspended in 6–7 mL SM buffer I (1 M NaCl, 8 mM MgSO_4_ · 7H_2_O, 25 mM Tris-HCl pH 7.5) and passed through a 0.2 μm pore size membrane filter before undergoing concentration and further purification in a CsCl gradient for 20 h at 150,000× *g* and 4 °C. The extracted phage band was then dialysed first in SM buffer I and then twice with SM buffer II (100 mM NaCl, 8 mM MgSO_4_ · 7H_2_O, 25 mM Tris-HCl pH 7.5) in order to remove the CsCl. The purified phage was then stored at 4 °C (see stability trials, Section 2.4). Bacteriophage samples stored in SM buffer II were used in human cell assays and stability trials. Samples were processed promptly due to their instability in SM buffer II.

#### 4.2.3. Plaque Assay—Quantification of Bacteriophages of Interest

Bacteriophage titre was determined via a soft agar plaque assay, using 0.7% agar top LBA [47]. One hundred microliters of serially diluted bacteriophage were incubated with 100 μL host cells (1 × 10^8^ CFU/mL) at room temperature for 15 min before 3 mL molten top agar was added and poured over a 90 mm 1.5% agar LBA plate. Plaques were quantified after overnight incubation at 37 °C. For spot/drop tests, 100 μL host cells (1 × 10^8^ CFU/mL) were added to molten 0.7% LBA, poured over a 1.5% agar LBA plate, and allowed to set before 5 μL bacteriophage (1 × 10^9^ PFU/mL) or 5 or 10 µL antibiotic were spotted onto the surface and allowed to dry.

#### 4.2.4. Classification of Bacteriophage Family

Bacteriophage families were classified as previously described, using electron microscopy [27,29,48].

### 4.3. Bacterial Host Characterisation

#### Antibiotic Susceptibility and MIC Testing

An initial screen of antibiotic susceptibilities was done using a drop test, where 10 µL of different antibiotics were spotted onto a lawn of *A. baumannii* on LBA. To determine if the three *A. baumannii* host strains were multi, extensively or pan drug resistant, the more quantitative disc diffusion method was carried out on selected clinically relevant antibiotics according to CLSI guidelines [49]. A further investigation into the colistin, apramycin, tigecycline, and meropenem resistance profiles of the *A. baumannii* host strains, was performed according to broth microdilution methods outlined by EUCAST [50] using cation adjusted Mueller Hinton broth 2 (CA-MHB) (Sigma-Aldrich—17.5 g/L acid hydrolysate of casein, 3 g/L beef extract, 1.5 g/L starch) and an initial inoculum of 5 × 10^5^ CFU/mL. The CA-MHB used with tigecycline was either used fresh (<12 h) or frozen and used later, in order to optimise the activity of this antibiotic, which can be negatively impacted by media that has been stored for too long [10].

### 4.4. Bacteriophage Activity against the Bacterial Host

#### 4.4.1. Twenty-Four-Hour *A. baumannii* Growth Curves

A total load of 1 × 10^7^ CFU *A. baumannii* hosts were added to each of the wells of a 96 well plate (5 × 10^7^ CFU/mL). Bacteriophages were added to a MOI of 0.01 (final load of 5 × 10^5^ CFU/mL) (total volume = 200 µL). Samples were grown in LB at 37 ± 2 °C shaking in a plate reader with measurements of the optical density (OD_600_) taken every five minutes over a 24-h period. Additional MOIs were also tested in the MOI optimisation growth curve assay (Supplementary Figure S3a–e).

#### 4.4.2. Triphenyl Tetrazolium Chloride Assay

After the 24 h growth curve, OD_600_ readings were completed, 50 µL of each sample was added to 150 µL water, and 50 µL 0.1% triphenyl tetrazolium chloride (TTC, Sigma-Aldrich) was diluted in LB and samples were incubated statically at 37 °C for three hours [48]. At this point the OD_540_ was measured, with the red coloured product being used as an indicator of relative metabolism. All tests were carried out with biological triplicates, with triplicate technical replicates assayed each time. Apramycin (final concentration of 500 µg/mL) was used as a positive control during this assay (a non-clinically relevant antibiotic was selected due to the host strains being resistant to most clinically relevant antibiotics trialled) (Supplementary Figure S4). A statistical analysis of results was carried out with Microsoft Excel using a two-tailed paired *t*-test, with a *p*-value of less than 0.05 being used as the threshold for significance.

### 4.5. Whole Genome Sequencing and Bioinformatics Analysis

Extraction and purification of bacteriophage genomic DNA was performed from on a high titre phage lysate (10^9^ PFU/ml) using a phenol:chloroform method as previously described [47]. An Illumina sequencing library was generated with the Nextera XT DNA library preparation kit, following the manufacturer’s instructions. Sequencing was performed on an Illumina MiSeq (250 bp paired-end). The resultant reads were trimmed with Sickle version 1.33 using default settings [51], prior to being assembled with SPAdes version 3.6.0 using the “--only-assembler” option [52]. The resulting single contig was annotated with Prokka version 1.11 using a custom database constructed from proteins of all current viral genomes [53]. The genome sequence of the selected bacteriophage was deposited in GenBank [33] under accession number MN864865. The annotated ORFs were searched by using BLASTn to detect virulence and antibiotic resistance genes in the virulence factor database (VFDB) [31] and antibiotic resistance genes database (ARDB) [32]. Hits with more than 75% coverage and 50% identity were considered as positive results.

To compare the vPhT2 with other bacteriophages, a BLASTn search with the vPhT2 genome against the NCBI database was performed and the most similar genomes were selected for further study [33]. The average nucleotide identity (ANI) percentages, based on BLAST pairwise sequence alignments, of these bacteriophage genomes were calculated using JSpeciesWS version 3.2.6 [54] with the default settings. An ANI of above 95% was classed as an indicator of two genome being from the same species [55]. The circular map of the bacteriophage genome was generated through the BLAST Ring Image Generator (BRIG) program, version 0.95, written by Alikhan et al., Australian Infectious Diseases Research Centre, School of Chemistry and Molecular Biosciences, The University of Queensland, Brisbane, QLD 4072, Australia [56].

### 4.6. Stability Trials

Aliquots of propagated bacteriophage were stored in the fridge (4 °C), at room temperature (21 °C, both protected and exposed to light), at 37 °C and in the freezer (−20 °C) in LB or SM buffer II for 14 days. Samples were taken after one, three, eight and 14 days storage and were quantified using the plaque assay described above and tested in duplicate. Aliquots stored in the freezer only underwent one freeze/thaw cycle each. Additional aliquots of samples suspended in LB were also kept in the fridge for 10 months to provide data on longer term storage in this location. Samples were plated in duplicate and reductions in titre were calculated as PFU/mL and log_10_PFU/mL compared to samples from time zero.

### 4.7. Bacteriophage Activity in the Human Cell Environment

#### 4.7.1. Endotoxin Testing

The presence of endotoxins in purified bacteriophage samples was measured using the Limulus Amebocyte Lysate (LAL) Chromogenic Endpoint Assay Kit from Hycult Biotech, according to the protocols supplied with the kit.

#### 4.7.2. Lactate Dehydrogenase Cytotoxicity Assay

Human cell lines for this study were T24 urinary bladder epithelial cells (ATCC^®^ HTB-4™) and hCMEC/D3 blood brain barrier endothelial cells (Sigma Aldrich SCC066). Cytotoxicity to human cells was measured using the Invitrogen™ CyQUANT™ LDH Cytotoxicity Assay Kit, according to the protocols supplied with the kit.

Human cell lines were grown up at 37 °C in their own cell specific media with 5% CO_2_ until they reached a suitable level of confluency. Cells were then trypsinised and resuspended in Leibovitz media (Sigma-Aldrich) with 5% FBS (Gibco) to a final concentration of 400,000 cells/mL. One millilitre of this suspension was then added to the wells of six well plates and grown overnight at 37 °C (no CO_2_ supplementation). On the same day, an overnight culture of *A. baumannii* was started in Leibovitz media + 5% FBS. Bacteriophages were diluted in LB to a final concentration of 1 × 10^9^ PFU/mL. After 24 h growth, 800 µL of *A. baumannii* overnight culture (with refreshed medium) or 800 µL Leibovitz medium + 5% FBS were added to the human cell lines and incubated at 37 °C for one hour. After an hour, 200 µL host specific bacteriophage or 200 µL LB medium was added to relevant samples (final bacteriophage concentration of 1 × 10^8^ PFU/mL). The total volume per well of the six well plates was 2 mL. Samples were then incubated for a further 23 h at 37 °C. After this incubation, aliquots were taken from each sample and the OD_600_ measured, as an indicator of *A. baumannii* turbidity. A second set of aliquots was taken in order to titre the CFU/mL of *A. baumannii* present, as a measure of host bacteria viability. A third set of aliquots was also taken and centrifuged at 17,000× *g* in a microfuge for five minutes to produce a cell pellet. Fifty microliters of the supernatant were then transferred to the wells of a 96 well plate in triplicate before adding 50 µL LDH assay test solution. Samples were mixed by tapping the side of the plate and then covered with foil to protect them from light during a 30-min incubation at room temperature. As a control, 200 µL lysis buffer supplied with the kit was added to relevant samples 45 min prior to adding LDH test solution. Fifty microliters of stop solution were added to all samples before measuring the optical density (OD) at 490 and 680 nm. Relative cytotoxicity to human cell lines was calculated from OD_490_ minus OD_680_. All tests were carried out with triplicate biological replicates. A statistical analysis of results was carried out with Microsoft Excel using a two-tailed un-paired *t*-test, with a *p*-value of less than 0.05 being used as the threshold for significance.

### 4.8. Synergy Trials

Synergy trials between colistin and bacteriophage vPhT2 were carried out using *A. baumannii* AB183 in a checker-board assay, following the same set up methods as the MIC testing, according to the EUCAST broth microdilution methods [50]. Synergy of colistin with the bacteriophages was determined from fractional inhibitory concentration (FIC) values, where a FIC is the MIC for an agent working in combination divided by the MIC of an agent when working alone. If the sum of the FIC values for both agents is ≤0.5, then this indicates synergy, whereas a ΣFIC > 0.5 and <2 shows indifference, and a value ≥2 indicates the antagonism of two agents against each other [39]. 

## 5. Conclusions

Understanding the in vivo activity of bacteriophages against their host bacteria is the first step in the successful development of a commercial or clinical product. In this study, the performance of a broad host range bacteriophage was analysed against its *A. baumannii* host and in the presence of human cell lines through the use of growth curves, bacterial viability assays, cytotoxicity studies, whole genome sequencing, stability testing, and assaying for synergy with colistin. Bacteriophage vPhT2 has shown potential to be made into a safe hand sanitizer or antimicrobial for use in hospitals, showing promise for development into a phage therapy tool in the future.

## Figures and Tables

**Table 1:** Overview of properties of *Acinetobacter baumannii* bacteriophages with broad host ranges.

Bacteriophage	Abbreviated Name	Previously Published Name	Host Strain	Family	Plaque Size
vB_AbaM_PhT2	vPhT2	ØAB02 [27,29]	*A. baumannii* AB183	*Myoviridae*	1 mm
vB_AbaM_PhT4	vPhT4	ØAB04 [27]	*A. baumannii* AB22	*Myoviridae*	12 mm
vB_AbaP_PhT29	vPhT29	ØAB29 [29]	*A. baumannii* AB20	*Podoviridae*	2–3 mm
vB_AbaP_PhT39	vPhT39	ØAB39 [29]	*A. baumannii* AB22	*Podoviridae*	12 mm
vB_AbaM_PhT44	vPhT44	ØAB44 [29]	*A. baumannii* AB20	*Myoviridae*	2–3 mm

See also Figures S1 and S2.

**Table 2:** Relative metabolic endpoint activity and turbidity of *Acinetobacter baumannii* samples treated for 24 h with bacteriophages or apramycin.

Sample	Relative Metabolic Activity (OD_540_) ^1^			Relative Turbidity (OD_600_)		
	Average (OD_540_ ± SD)	Normalised (% ± SD)	Significance of Results ^2^	Average (OD_600_ ± SD)	Normalised (% ± SD)	Significance of Results ^2^
AB20	1.579 ± 0.422	100.00 ± 0.00	N/A	1.863 ± 0.264	100.00 ± 0.00	N/A
AB20 + apramycin	0.006 ± 0.001	0.41 ± 0.110	***	0.240 ± 0.144	13.47 ± 8.57	***
AB20 + vPhT29	1.030 ± 0.368	64.89 ± 11.28	***	1.055 ± 0.338	56.87 ± 16.74	***
AB20 + vPhT44	1.027 ± 0.326	65.22 ± 11.15	***	1.119 ± 0.319	59.53 ± 11.51	***
AB20 + vPhT29 + vPhT44	1.151 ± 0.443	71.52 ± 13.82	**	1.156 ± 0.262	60.84 ± 8.07	***
AB22	1.520 ± 0.188	100.00 ± 0.00	N/A	1.811 ± 0.127	100.00 ± 0.00	N/A
AB22 + apramycin	0.009 ± 0.003	0.60 ± 0.28	***	0.245 ± 0.127	13.56 ± 7.35	***
AB22 + vPhT4	0.888 ± 0.151	58.96 ± 11.08	***	0.592 ± 0.170	32.96 ± 10.01	***
AB22 + vPhT39	0.984 ± 0.198	64.82 ± 11.28	***	0.695 ± 0.240	38.60 ± 13.92	***
AB22 + vPhT4 + vPhT39	0.794 ± 0.186	56.19 ± 12.42	*	0.673 ± 0.165	38.16 ± 9.67	**
AB183	1.562 ± 0.186	100.00 ± 0.00	N/A	1.671 ± 0.071	100.00 ± 0.00	N/A
AB183 + apramycin	0.009 ± 0.007	0.560 ± 0.370	***	0.134 ± 0.061	7.93 ± 3.39	***
AB183 + vPhT2	0.978 ± 0.306	62.94 ± 20.13	**	0.832 ± 0.247	49.79 ± 14.19	***

* *p* < 0.05, ** *p* < 0.01, *** *p* < 0.001, SD = standard deviation, N/A = not applicable. Apramycin concentration = 500 µg/mL. ^1^ Relative metabolic activity was calculated from OD_540_ readings after application of triphenyl tetrazolium chloride (TTC). ^2^ Significance was calculated from a two tailed paired *t*-test compared to the untreated, bacteria only controls.

**Table 3:** Ranking of bacteriophage efficacy and performance across all assays from this study.

Variable	vPhT2	vPhT4	vPhT29	vPhT39	vPhT44
Host range ^1^	1	4	4	3	2
Host metabolism ^2^	2	1	4	3	5
Host turbidity ^3^	3	1	4	2	5
Maintaining low host turbidity ^4^	1	2	3	3	5

Bacteriophages that performed equivalently have been given the same ranking for that variable. ^1^ Host range (1 = broadest host range, 5 = narrowest host range). ^2^ Reduction in relative endpoint host metabolism (OD_540_ of TTC assay) after 24 h incubation with the bacteriophage (1 = lowest metabolism, 5 = highest metabolism). ^3^ Reduction in endpoint host turbidity (OD_600_) after 24 h incubation with the bacteriophage (1 = lowest turbidity, 5 = highest turbidity). ^4^ Length of time maintaining a low host OD_600_ during 24-h growth curves (1 = longest, 5 = shortest).

**Table 4:** Comparison between vPhT2 and its most similar genomes in the NCBI database including pairwise genome comparison ANI-tree similarity matrix compare and gene structure of vPhT2.

Characteristic	vPhT2	AbTZA1	AM101	KARL-1	vB_ApiM_fHyAci03	ZZ1
vPhT2 ANI (%)	N/A	95.34	78.16	77.45	77.35	71.31
Accession number	MN864865	MK278860	MH165274	MH713599	MH460829	HQ698922
Genome size (bp)	166,330	168,223	166,487	166,560	165,975	166,687
GC content (%)	36.4	36.3	36.7	36.8	36.8	34.4
Number of ORFs	246	253	250	253	247	256
Number of tRNAs	9	6	8	0	8	8
Year uploaded	2019	2019	2019	2018	2018	2012
Isolate location	Thailand	Israel	Russia	Germany	Finland	China
Family	*Myoviridae*	*Myoviridae*	*Myoviridae*	*Myoviridae*	*Myoviridae*	*Myoviridae*
Reference	This study	Unpublished	Unpublished	Jansen et al. [34]	Pulkkinen et al. [35]	Jin et al. [36]

ANI = average nucleotide identity, N/A = not applicable, bp = base pairs, ORF = open reading frame.

**Table 5:** Logarithmic reduction in bacteriophage vPhT2 after storage in SM buffer II or LB for 14 days in different temperature and light conditions.

Storage Location	Storage Reagent	Reduction in Titre (log_10_PFU/mL ± SD)			
		1 Day	3 Days	8 Days	14 Days
Fridge (4 °C)	SMII	NR	0.66 ± 0.00	1.57 ± 0.03	2.60 ± 0.29
Room temperature (21 °C)	SMII	0.32 ± 0.01	0.79 ± 0.18	1.87 ± 0.04	2.94 ± 0.33
Room temperature with light (21 °C)	SMII	0.32 ± 0.04	0.68 ± 0.03	1.92 ± 0.21	2.75 ± 0.10
Freezer (−20 °C)	SMII	1.99 ± 0.09	2.74 ± 0.01	3.59 ± 0.18	4.23 ± 0.06
Biological incubator (37 °C)	SMII	0.79 ± 0.14	1.58 ± 0.06	3.88 ± 0.07	4.88 ± 0.21
Fridge (4 °C)	LB	NR	NR	NR	NR
Room temperature (21 °C)	LB	NR	NR	NR	NR
Room temperature with light (21 °C)	LB	NR	NR	NR	NR

PFU = plaque forming units, SD = standard deviation, NR = no significant reduction (*p* > 0.05), LB = lysogeny broth, SMII = SM buffer II (see methods).

**Table 6:** Data on units of endotoxins measured in bacteriophage preparations.

Sample	Sample Concentration	EU/mL
Crude vPhT2 in LB	5 × 10^5^ PFU/mL	Above threshold of standard curve
Purified vPhT2 in SM buffer II	1 × 10^8^ PFU/mL	10.92
LB medium	N/A	0.09
SM buffer II	N/A	0.03
Leibovitz medium	N/A	0.05

PFU = plaque forming units, EU = endotoxin unit, LB = lysogeny broth.

**Table 7:** Raw data for lactate dehydrogenase levels (OD_490_–OD_680_) released by different human cell lines treated with *Acinetobacter baumannii* or vPhT2 compared to no treatment.

Human Cell Line	Average Relative LDH Release (OD_490–680_ ± SD)		
	Human Cells Only	Human Cells and vPhT2	Human Cells and AB183
T24	0.185 ± 0.012	0.204 ± 0.011	2.444 ± 0.057
hCMEC/D3	0.017 ± 0.002	0.022 ± 0.004	0.486 ± 0.125

LDH = lactate dehydrogenase, SD = standard deviation.

**Table 8:** Raw data for *Acinetobacter baumannii* turbidity (OD_600_) and titre (log_10_CFU/mL) from samples grown in the presence of human cell lines.

Human Cell Line	Average OD_600_ (±SD)		Average log_10_CFU/mL (±SD)	
	Human Cells and AB183	Human Cells, AB183 and vPhT2	Human Cells and AB183	Human Cells, AB183 and vPhT2
T24	1.051 ± 0.040	0.156 ± 0.051	9.47 ± 0.01	6.13 ± 0.11
hCMEC/D3	0.989 ± 0.073	0.105 ± 0.017	9.46 ± 0.07	6.38 ± 0.13

CFU = colony forming units, SD = standard deviation.

**Table 9:** Susceptibility of *Acinetobacter baumannii* host strain AB183 to colistin in combination with bacteriophage vPhT2.

Treatment	Independent MIC Value	Combined MIC Value	FIC Values
Colistin	1 µg/mL	0.25 µg/mL	0.25
vPhT2	500 PFU/mL	50 PFU/mL	0.1
FICI			0.35

MIC = minimum inhibitory concentration, FIC = fractional inhibitory concentration, FICI = fractional inhibitory concentration index (calculated FICI = combined MIC/independent MIC), PFU = plaque forming units.

## Supplementary Materials

The following are available online at https://www.mdpi.com/2079-6382/9/4/200/s1, **Figure S1**: Morphology of plaques formed by bacteriophages on a lawn of *Acinetobacter baumannii*. **Figure S2**: Transmission Electron Micrographs of *A. baumannii* bacteriophages. **Table S1**: Details on clinical sources of *Acinetobacter baumannii* host strains. **Figure S3a–e**: Eight-hour growth curve for Acinetobacter baumannii strains grown with bacteriophages added at different multiplicity of infections (MOIs). **Table S2**: Logarithmic titer of bacteriophage vPhT2 after storage for 30 days in different temperature and light conditions. **Table S3**: Minimum inhibitory concentrations (MICs) of antibiotics against *Acinetobacter baumannii* host strains. **Figure S4**: Photo of drop test screen for zones of inhibition caused by a range of antibiotics against *Acinetobacter baumannii* strains. **Supplement File**: Selected-MDRAB-150-isolates.
