# Supplementary material for: Investigating Bacteriophages Targeting the Opportunistic Pathogen Acinetobacter baumannii
Source: Antibiotics (Basel). 2020 Apr 22;9(4):200. doi: 10.3390/antibiotics9040200 (PMC7235909; doi:10.3390/antibiotics9040200)
Supplement: Supplementary file 1 [file antibiotics-09-00200-s001.zip › supp/antibiotics-774160-supp.pdf]

antibiotics

MDPI

Supplementary Materials

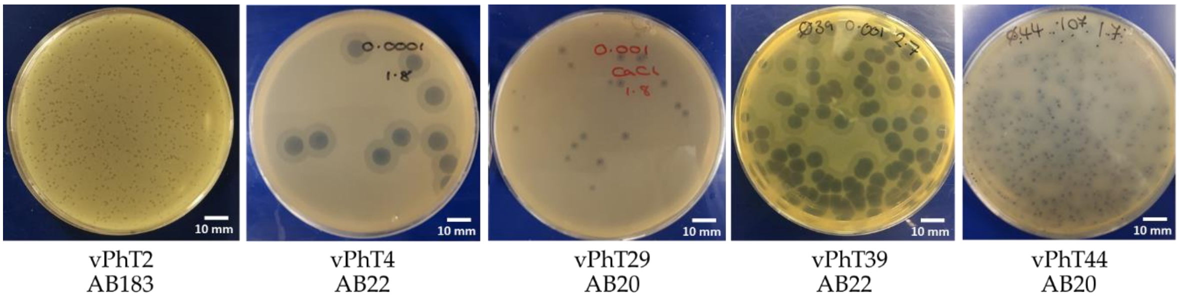

Figure S1. Morphology of plaques formed by bacteriophages on a lawn of *Acinetobacter baumannii* hosts after overnight incubation.

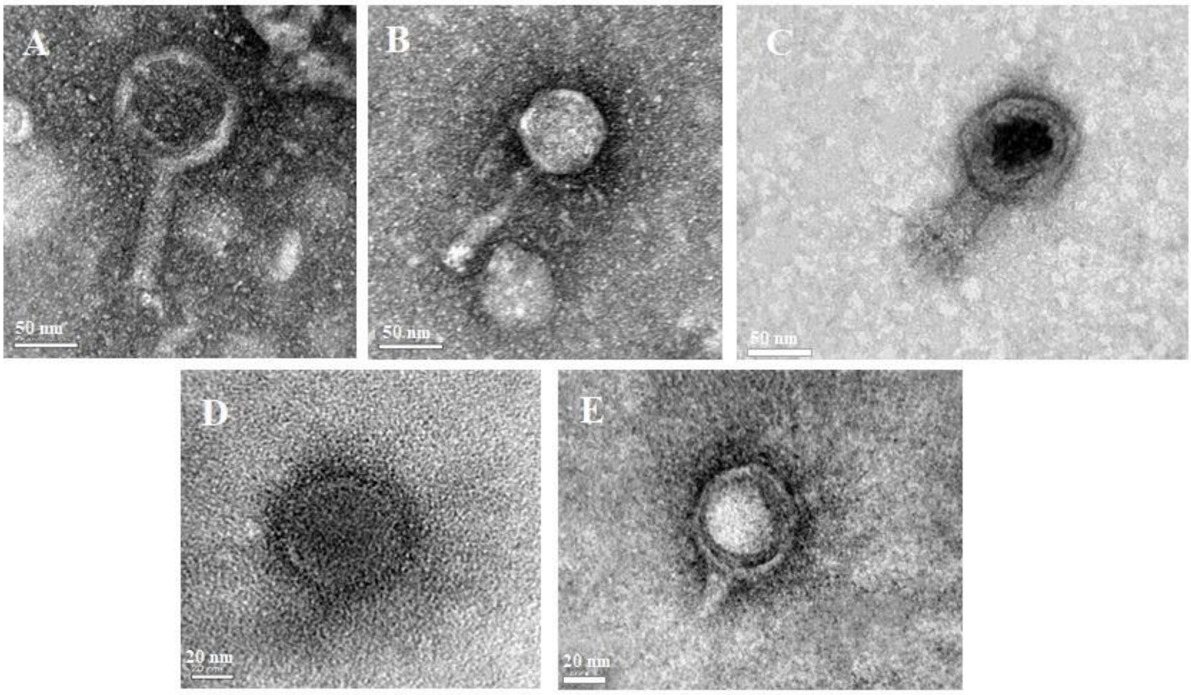

Figure S2: Transmission Electron Micrographs of *A. baumannii* bacteriophages. (**A**) vPhT2 (previous name ØABP02), (**B**) vPhT4 (previous name ØABP04) and (**C**) vPhT44 (previous name ØABP44) belonging to the *Myoviridae* family. (**D**) vPhT29 (previous name ØABP29) and (**E**) vPhT39 (previous name ØABP39) belonging to the *Podoviridae* family. The phages were negatively stained with 2% uranyl-acetate. Bar corresponds to 50 nm. (A, B, C) and 20 nm. (D, E).

Table S1: Details on clinical sources of *Acinetobacter baumannii* host strains.

A. baumannii host strain	Hospital location	Number of beds	Isolation year	Specimen type	Ward type
AB20	Central Thailand (HA)	653	2013	Sputum	ICU
AB22	Central Thailand (HA)	653	2013	Wound	General
AB183	Northern Thailand (HC)	756	2014	Sputum	ICU

(ICU = intensive care unit).

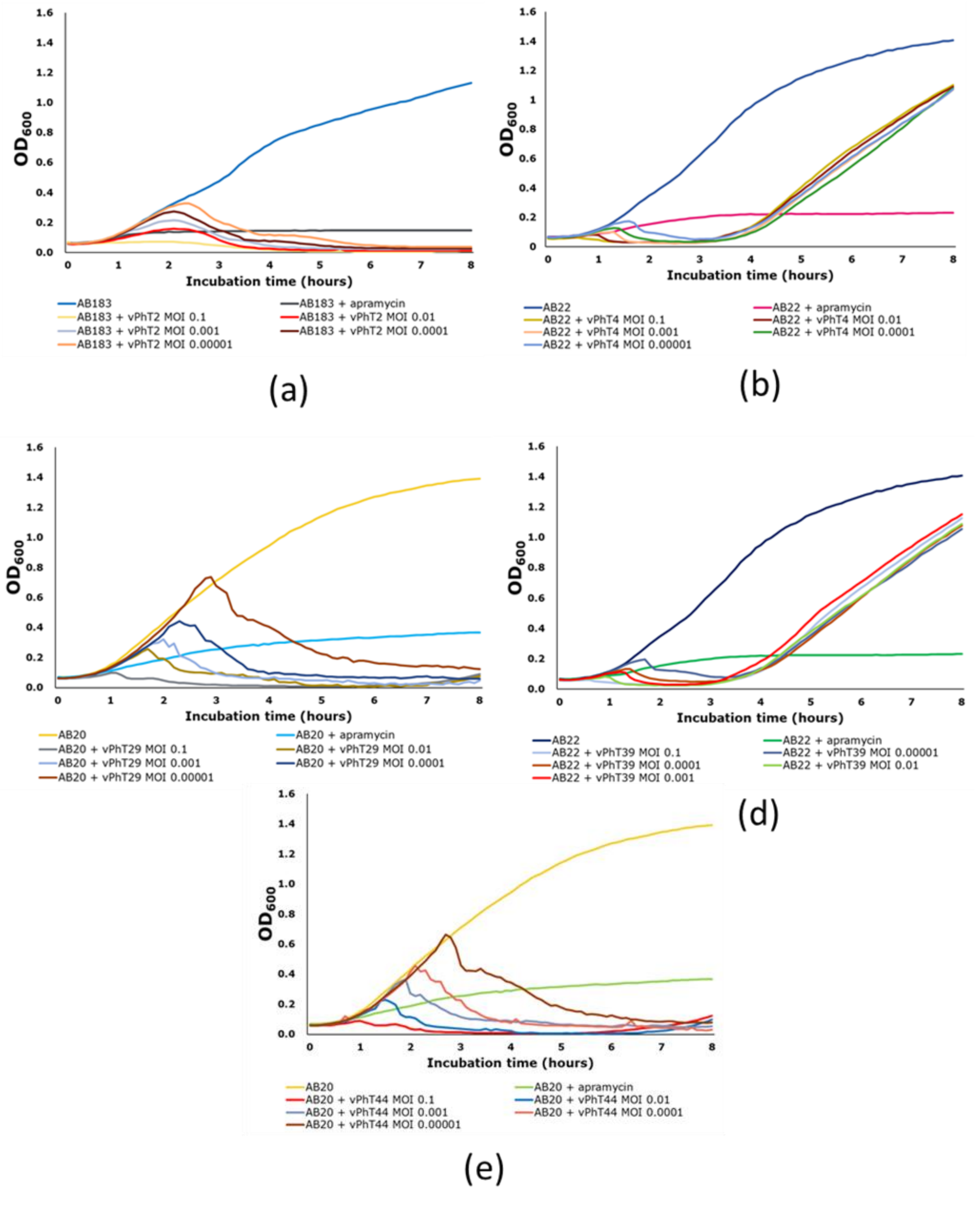

Figure S3a-e. Eight-hour growth curve for *Acinetobacter baumannii* strains grown with bacteriophages added at different multiplicity of infections (MOIs). Data displayed as OD_{600} (N=3). (a) vPhT2, (b) vPhT4, (c) vPhT29, (d) vPhT39, (e) vPhT44.

Table S2. Logarithmic titre of bacteriophage vPhT2 after storage in SM buffer II for 14 days in different temperature and light conditions.

Storage location	Bacteriophage recovery at each time point ($\log_{10}$PFU/mL ± SD)				
	**0 days**	**1 day**	**3 days**	**8 days**	**14 days**
Fridge (4 °C)	10.13 ± 0.15	10.03 ± 0.25	9.46 ± 0.00	8.55 ± 0.06	7.53 ± 0.61
Room temperature (21 °C)	N/A	9.80 ± 0.03	9.33 ± 0.41	8.26 ± 0.10	7.18 ± 0.69
Room temperature (21 °C) with light	N/A	9.81 ± 0.10	9.45 ± 0.06	8.20 ± 0.46	7.38 ± 0.22
Freezer (-20 °C)	N/A	8.14 ± 0.20	7.39 ± 0.03	6.54 ± 0.41	5.90 ± 0.14
Biological incubator (37 °C)	N/A	9.33 ± 0.32	8.55 ± 0.14	6.25 ± 0.17	5.24 ± 0.46

N/A = not applicable. PFU = plaque forming units. SD = standard deviation.

Table S3: Minimum inhibitory concentrations (MICs) of antibiotics against *Acinetobacter baumannii* host strains, as established from a broth microdilution method.

Antibiotic	MIC value (µg/mL)			Susceptibility cut off[1]	Resistance cut off[1]
	**AB20**	**AB22**	**AB183**	**≤ µg/mL**	**> µg/mL**
Meropenem	32	32	256	2	8
Colistin	1	2	1	2	2
Apramycin	16	16	32	No data	No data
Tigecycline	1	2	1	No data	No data

[1]Clinical breakpoints are taken from those defined by the European Committee on Antimicrobial Susceptibility Testing (EUCAST)[1,2].

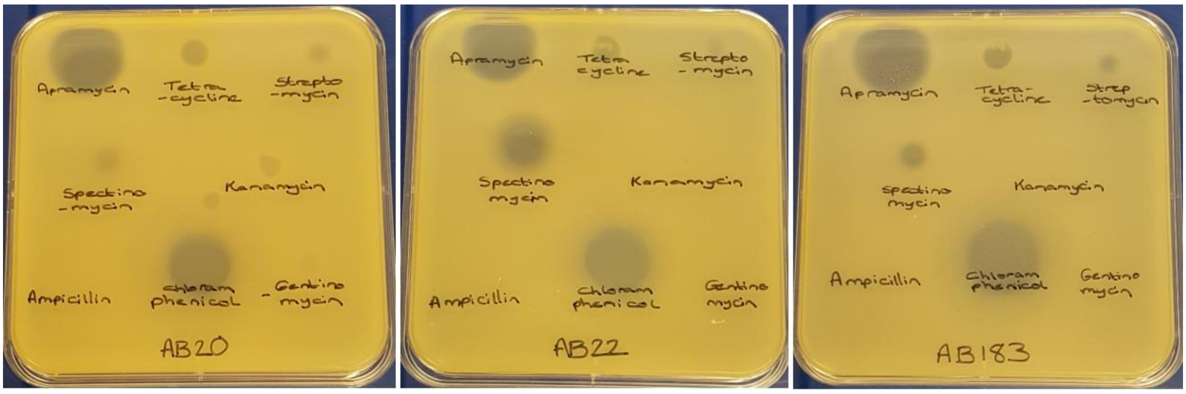

Figure S4: Photo of drop test screen for zones of inhibition caused by a range of antibiotics against *Acinetobacter baumannii* strains.

References

1. Hasselmann, C. Determination of minimum inhibitory concentrations (MICs) of antibacterial agents by broth dilution. *Clin. Microbiol. Infect.* **2003**, *9*, ix–xv.

2. EUCAST The European Committee on Antimicrobial Susceptibility Testing. Breakpoint tables for interpretation of MICs and zone diameters. Version 9.0, 2019. Available online: http://www.eucast.org (accessed on Sep 9, 2019).
